# Ectopia Cordis as a Lethal Neonatal Condition: A Case Report from Bahrain and a Literature Review

**DOI:** 10.1155/2022/6850305

**Published:** 2022-08-22

**Authors:** Shereen M. Mohamed, Hasan M. Isa, Amarjit K. Sandhu

**Affiliations:** ^1^Obstetrics and Gynaecology, Salmaniya Medical Complex, Manama, Bahrain; ^2^Arabian Gulf University, Pediatric Department, Salmaniya Medical Complex, Manama, Bahrain; ^3^Arabian Gulf University, Obstetrics and Gynaecology, Salmaniya Medical Complex, Manama, Bahrain

## Abstract

Ectopia cordis is a rare type of malformation where the heart is not located normally. It may be partially or completely located outside the thoracic cavity and can be associated with other congenital abnormalities. It results from failure of maturation of midline mesoderm and ventral body formation during embryogenic formation. The exact etiology remains unknown. The literature review reveals the prognosis for infants with ectopia cordis is very poor. Here, we are reporting the first case of a fetus that was prenatally diagnosed with ectopia cordis that was associated with omphalocele documented in our country. Considering the poor prognosis for the fetus, conservative management during the prenatal period was chosen.

## 1. Introduction

Ectopia cordis is a rare congenital malformation with an estimated incidence of 5.5 to 7.9 per million live births [1]. Haller published the first reported case in the year 1706, and it was studied further into different types by Weese in 1818 and Todd in 1836 [2]. It accounts for only 0.1% of all congenital heart diseases. It is a malformation in which the heart is abnormally located in the extra-thoracic position and may be isolated or associated with other anomalies such as omphalocele, congenital heart disease, or integrating Cantrell syndrome. The size and location of the defect influence the prognosis. Due to the rarity and rapid postpartum mortality, limited treatment options are known. The mortality rate remains very high [1, 3, 4]. Here, we are reporting a case of ectopia cordis in a four-day-old neonate.

## 2. Case Presentation

This is a four-day-old male neonate of a 23-year-old gravida 4, para 1 plus 2 previous abortions. The mother of the patient had a healthy female full-term baby who was delivered by caesarian section for fetal distress.

**Figure 1:**
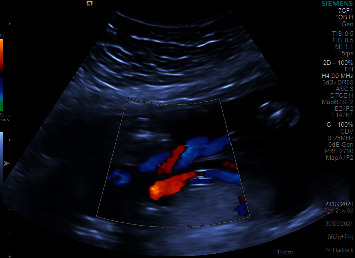
Ultrasound anomaly scan of a fetus at 21 weeks and six days of gestation with ectopia cordis showing that the heart lies completely outside the chest cavity (arrow).

**Figure 2:**
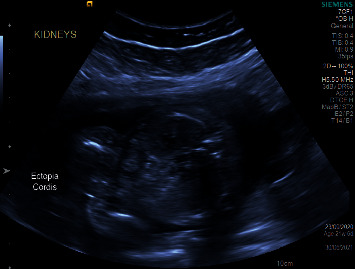
Ultrasound image showing both the kidneys and ectopia cordis.

**Figure 3:**
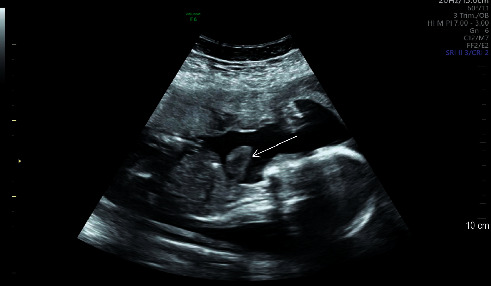
The heart is outside the chest cavity in this ultrasound image of a sagittal view (arrow), making it impossible to assess the nasal bone.

During pregnancy, the mother had a routine anomaly scan at 21 weeks and 6 days of gestation which showed ectopia cordis with a small chest and lung volume. The heart was lying completely outside of the chest cavity, with a normal four-chamber view and a regular heart rate and rhythm (Video 1). The outflow tract view was difficult to obtain due to restrictions of the examination. Antenatal ultrasound scan of the anomaly is shown in Figure 1. In the abdomen, the stomach bubble was present, the diaphragm was grossly intact, the liver was difficult to visualize, and the portal vein was not clearly visualized. Normal fetal umbilical cord insertion was noted. There was some protrusion above the level of the umbilical cord insertion site that might represent the liver or small bowel. Both kidneys and urinary bladder were normal during the sonography (Figure 2). The upper and lower extremities also appeared to be normal. Due to the fetus's location, the fetal nasal bone could not be assessed as shown in the sagittal view (Figure 3). The patient was followed up prior in a different health care institute and then presented to our hospital in the late second trimester which was 26 weeks and 6 days with her all-antenatal records. Her next follow-up visit to the clinic where she presented late was at 34 weeks and 6 days of gestation. Then, at 35 weeks of gestation, the mother had the first fetal echocardiogram at our hospital which confirmed that most of the cardiac mass was still outside the thoracic cavity with compression of great arteries upon entry to the chest with no other associated congenital anomalies. The right atrium, right ventricle, left atrium, mitral valve, left ventricle, and ventricular septum were all normal, and there was no pericardial effusion. The aorta was not well seen in view of the distortion of the anatomy.

During the pregnancy, there was no history of ingestion of unprescribed medications, use of illicit drugs, cigarette smoking, alcohol abuse, or history of chronic ill health. There was no relevant family history of congenital anomalies, genetic abnormalities, or history of an ectopia cordis in other family members. However, there was a history of first-degree consanguinity.

Despite an unfavorable prognosis for the fetus that has been explained to the mother, a conservative management during the prenatal care was planned upon the parent's request.

**Figure 4:**
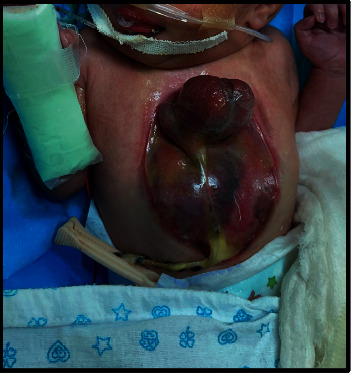
A newborn with ectopia cordis (the heart is lying outside the chest cavity with an abdominal wall defect suggestive of omphalocele).

At 36 weeks of gestation, she presented with scar tenderness and had an emergency lower segment caesarian section with an outcome of alive male baby born with an Apgar score of 9 at 1 minute and 10 at 5 minutes. His birth weight was 2.200 kilograms. The physical examination revealed a responsive newborn with an externalized heart totally outside the chest, without pericardium protection. The dynamic movement of heart beats outside the chest cavity is shown in the supplementary files (Video 2). In addition, an abdominal wall defect was detected suggesting an associated omphalocele (Figure 4). The remainder of the physical examination was normal. After birth, the infant's heart was covered with a warm saline-soaked sterile dressing and immediately transferred to the specialized neonate intensive care unit. Cardiac ultrasound could not be performed. Fetal karyotyping was performed, and results showed a male chromosomal complement with structural chromosomal abnormalities suggestive of chromosomal mosaicism. Comparative genomic hybridization (CGH) was recommended which could not be performed as the results of fetal karyotype were available only after the newborn expired. Hematological tests of newborns were normal. The newborn expired on the fourth postnatal day. Each couple was debriefed, and genetic counseling was provided regarding the maternal history of two previous abortions and first-degree consanguinity.

## 3. Discussion

Ectopia cordis is a congenital heart exposure which means the heart outside the thoracic cavity could be partial or complete [3]. The cause of this pathology is unknown. It can be due to failure of both lateral mesoderm development by the third week of the embryo formation and midline fusion of the chest wall during intrauterine life. This can lead to compression of the thorax resulting in rupture of the chorion or yolk sac at around the gestational age of 21 days [2]. Kim et al. and Dobell et al. classified ectopia cordis into five different types: (i) cervical: the heart is located in the neck with the sternum usually intact; (ii) thoracocervical: the upper portion of the sternum is split, and the heart is partially in the cervical region; (iii) thoracic: the sternum is split or absent, and the heart is partially or completely outside the thorax; (iv) thoracoabdominal: it usually accompanies Cantrell syndrome; (v) abdominal: where the heart passes through a defect in the diaphragm to enter the abdominal cavity [5, 6].

Ectopia cordis can be associated with chromosomal abnormalities like turner syndrome (XO), trisomy 21, and Edward syndrome (trisomy 18) [2, 7]. Intracardiac defects can also occur with ectopia cordis such as atrial septal defect (ASD), ventricular septal defect (VSD), tetralogy of Fallot (TOF), tricuspid atresia (TA), and double outlet right ventricular (DORV) [1, 8, 9].

Moreover, noncardiac malformations can occur such as pentalogy of Cantrell, omphalocele, diagrammatic hernia, hypoplastic lung disease, skeletal dysplasia, and cleft palate [2, 3, 10].

Despite that our patient had normal chromosomal study, an associated omphalocele was noted.

Differential diagnosis of ectopia cordis includes pentalogy of Cantrell, Beckwith–Wiedemann syndrome, amniotic band syndrome, and limb-body-wall complex [3].

Ectopia cordis is associated with the presence of amniotic band syndrome. Bands may be attached to the ectopic heart or a defect in the thorax and abdominal wall. Almost all cases of amniotic band syndrome are sporadic, and the risk of recurrence is low in subsequent pregnancy [11, 12].

Prenatal diagnosis has an important role in identifying ectopia cordis and its associated abnormalities. It can be detected in the first trimester or the beginning of the second trimester [13]. Unfortunately, in our case, it was diagnosed at 21 weeks and 6 days of gestation as the patient's mother presented to the hospital during the midsecondtrimester. Nonetheless, antenatal diagnosis is possible as early as first trimester using ultrasound. Almost 90% of associated defects can be detected. However, the three-dimensional ultrasound is better for scanning to visualize fetal bones due to its greater contrast compared to two-dimensional ultrasound [2, 14–18].

Fetal cardiac magnetic resonance imaging (MRI) is an alternative imaging option to echocardiography, where it was limited by maternal or fetal factors such as maternal obesity or abnormal fetal positions and placental calcifications [13, 16].

Chromosomal analysis is highly recommended due to its association with chromosomal abnormalities [13, 16].

Treatment of ectopia cordis requires a multidisciplinary approach consisting of a perinatologist, neonatologist, radiologist, pediatric, and pediatric cardiac surgeon. The multisurgical approach requires repair of omphalocele, sternal, diaphragmatic, pericardial defects, repair of intracardiac anomalies, and reducing the heart into the chest cavity. Since it is known that the thorax is small in ectopia cordis, the surgical approach is challenging and reducing the heart can cause kinking and compression of vessels [19]. The first trial of ectopia cordis repair was performed in 1925 by Cutler and Wilens [20], followed by the Koop trial in 1975 which achieved the first successful repair of thoracic ectopia cordis in two stages [21]. In 1995, Amato et al. achieved a successful single-stage repair of thoracic ectopia cordis [22]. Due to the rarity of ectopia cordis, there is no fixed technique used. However, the surgical approach includes closure of the chest wall and sternal defects, repair of omphalocele, placement of the heart into the thorax, and repair of intracardiac defects [14, 19, 23].

The efficacy of surgery depends on the degree of ectopia cordis and other congenital heart defects. The main aim of the surgical treatment is to provide a soft tissue cover to the heart along with reduction of the heart to the thoracic cavity and reconstruction of the chest wall [18, 24].

In the year of 2020 in Indonesia, Limanto and Soebroto did a palliative surgery on a five-day-old full-term newborn for a complete thoracic ectopia cordis with the aim of covering the exposed heart with the use of bovine pericardium material that is commonly used in reconstruction in cardiac surgery. Unfortunately, the patient expired on the third postoperative day due to heart failure [25]. In the year 2021, Yıldız et al. in Turkey reported four cases of ectopia cordis with abdominal, thoracoabdominal, partial thoracoabdominal, and middle sternum defects that were treated surgically. They concluded that the degree and level of exposure of the heart preclude initial survival, and complex repairs of intracardiac defects may be difficult in cases requiring shunts for long-term palliation [26]. In the same year in Iraq, Aboud et al. reported a case of complete thoracic ectopia cordis, but due to poor general condition, the newborn expired on 36 hours of life before any intervention [27]. Another case of thoracoabdominal ectopia cordis from Tanzania was reported by Lodhia et al. but in view of the lack of a cardiac center and waiting for relocating the patient to a cardiac institute, the newborn had a cardiac arrest on the fifth day of life and surgery of relocating the heart was not carried out [28]. Lastly, in the same year, Kebalo et al. in the Togolese Republic reported a case of midsternal defect of ectopia cordis and the newborn expired 22 hours after birth which also made them unable to perform any procedure [29]. The most recent case reported in the year 2022 from Saudi Arabia by Alshamiri et al. was a case of incomplete ectopia cordis in a 23-year-old male, who expired due to COVID-19 infection [30]. This last patient might indicate that in patients with incomplete or partial ectopia cordis, survival may be possible.

Unfortunately, no surgical intervention could be carried out in our case because the pediatric surgeon believed that the thoracic cavity volume was very small.

The overall prognosis of ectopia cordis is poor as it is a lethal anomaly, and despite advances in neonatal cardiac surgery, this remains a surgical challenge with only a few long-term survivals.

In conclusion, the management of ectopia cordis is a challenge for any obstetrician and pediatric surgeon due to its rarity. It causes mental agony and anxiety to the parents. With early diagnosis, termination of pregnancy can be one of the options. Once it is diagnosed late, it needs a multidisciplinary approach, and the services of a very experienced pediatric surgeon to improve the prognosis.

## Data Availability

The data supporting the findings of this study are available within the article. Raw data that supports the findings of this study are available from the corresponding author, upon reasonable request.

## Supplementary Materials

Supplementary Materials There is an ultrasound video before birth and a video of the newborn right after birth showing the dynamics of the heartbeat outside the thoracic cavity uploaded as a zip file in supplementary files.
